# Should prophylactic antibiotics be used routinely in epistaxis patients with nasal packs?

**DOI:** 10.1308/003588413X13511609954734

**Published:** 2013-01

**Authors:** TC Biggs, K Nightingale, NN Patel, RJ Salib

**Affiliations:** University Hospital Southampton NHS Foundation Trust,UK

**Keywords:** Epistaxis, Guidelines, Antibiotics, Audit, Nasal, Packing

## Abstract

**Introduction:**

The current mainstream practice in otolaryngology departments relating to the use of prophylactic antibiotics in epistaxis patients requiring nasal packing is highly variable. This is due primarily to the lack of any validated guidelines. As such, we introduced a new treatment algorithm resulting in significant reduction of use in the systemic antibiotics, with emphasis instead on the use of topical antibiotics. The results were validated through a complete audit cycle.

**Methods:**

A total of 57 patients undergoing nasal packing for spontaneous epistaxis were studied. Reaudit occurred after the implementation of new guidelines. Telephone surveys were conducted six weeks after hospital discharge, assessing infective nasal symptoms as well as rebleeding and readmission rates.

**Results:**

Systemic antibiotic prescribing in anterior nasal packing fell by 58.2% between audit cycles with no statistically significant associated increase in infective nasal symptoms, rebleeding or readmission rates six weeks following hospital discharge.

**Conclusions:**

Systemic prophylactic antibiotics are unnecessary in the majority of epistaxis patients with nasal packs. The use of topical antibiotics such as Naseptin^®^ may be more appropriate, cheaper and as effective. Implementation of this treatment algorithm will help standardise systemic antibiotic usage in epistaxis patients with nasal packing and should reduce costs associated with unnecessary use of such medication.

Although it is generally accepted that the use of prophylactic systemic antibiotics in spontaneous epistaxis patients with posterior nasal packing is indicated, the equivalent practice in anterior nasal packing remains highly variable across the National Health Service (NHS).^1,2^ Some ear, nose and throat (ENT) departments use systemic prophylactic antibiotics routinely in the context of anterior nasal packing for spontaneous epistaxis while others use no form of antibiotic cover at all.^2^ The ENT department (tertiary referral centre) at the University Hospital Southampton NHS Foundation Trust (UHSFT) had no existing guidelines for prophylactic antibiotic use in nasal packing, with the majority of patients (both anterior and posterior packs) receiving some form of systemic antibiotic cover. This two-cycle audit outlines the change from this practice to that of selective prescribing and alternative use of topical antibiotics, with its resultant effects on nasal symptoms (pain, crusting, nasal discharge), rebleeding and readmission rates at six weeks following hospital discharge.

**Figure 1:**
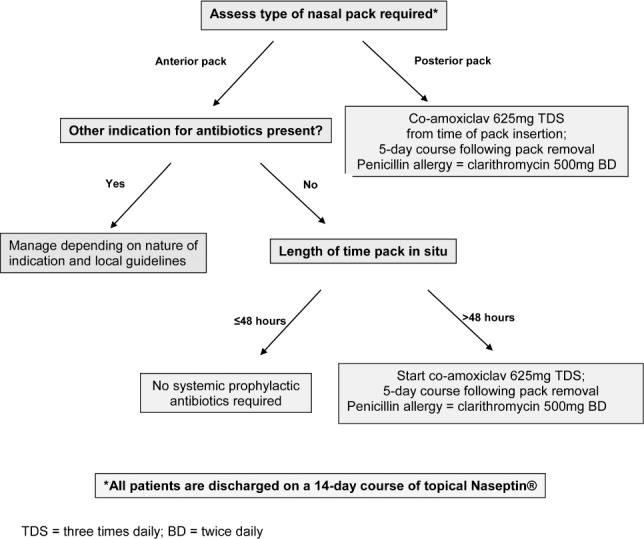
Treatment algorithm for the use of prophylactic antibiotics in epistaxis patients with nasal packing

## Methods

Overall, 57 consecutive patients admitted to the UHSFT ENT department with spontaneous epistaxis requiring anterior nasal packing were included in the study. In the ENT department, the first line management of patients presenting with epistaxis is to undertake nasal cautery where possible, with nasal packing reserved for those patients where cautery fails or where the bleeding is too severe to control with this technique.

The first cycle included 38 patients presenting between 30 September 2010 and 11 January 2011. The data were collected retrospectively through examination of the inpatient notes. They included timing of nasal packing, type of packing, use and timings of antibiotics including oral and topical (Naseptin^®^; Alliance, Chippenham, UK), timing of nasal packing removal and presence of co-existing medical conditions requiring antibiotic use. Patients were followed up six weeks after hospital discharge using a telephone survey for assessment of infective nasal symptoms and recurrent epistaxis necessitating readmission to hospital. Only patients with complete data sets were included in the study. There were a number of patients admitted during the study period who did not have complete accurate information in their inpatient notes or could not be contacted following discharge, resulting in exclusion. This impacted on the final numbers included in the study.

During the first cycle of the audit, it was common practice at UHSFT to prescribe systemic prophylactic antibiotics in all nasally packed epistaxis patients. After analysis of the first cycle and following agreement with consultants of the ENT department as well as guidance from the microbiology department, a new treatment algorithm was drafted (Fig 1). The guidelines were implemented with dissemination to the junior doctors detailing the use of prophylactic antibiotics in spontaneous epistaxis patients requiring nasal packing.

The second cycle using the new guidelines included 19 patients studied between 14 June 2011 and 17 August 2011. As in the first audit cycle, patients were contacted via a telephone survey six weeks following discharge from hospital.

Results were collated and analysed in Excel^®^ 2009 (Microsoft, Redmond, WA, US) with statistical analysis performed using SPSS^®^ version 20 (SPSS, Chicago, IL, US). The data were non-parametric and Fisher’s exact test was therefore undertaken to conduct the statistical analysis (significance achieved at *p*<0.05).

**Table 1:** Results of the six-week follow-up telephone questionnaire

	First cycle (*n*=38)	Second cycle (*n*=19)	*p*-value (Fisher’s exact test)
Nasal discharge	2 (5.3%)	0 (0%)	0.548
Nasal crusting	3 (7.9%)	0 (0%)	0.544
Nasal pain	2 (5.3%)	1 (5.3%)	0.999
Sinusitis	1 (2.6%)	1 (5.3%)	0.999
Chest infection	2 (5.3)	0 (0%)	0.548
Rebleeding	9 (23.7%)	2 (10.5%)	0.304
Readmission	3 (7.9%)	0 (0%)	0.544
Overall complications	22/266 (8.3%)	4/133 (3.0%)	

**Figure 2:**
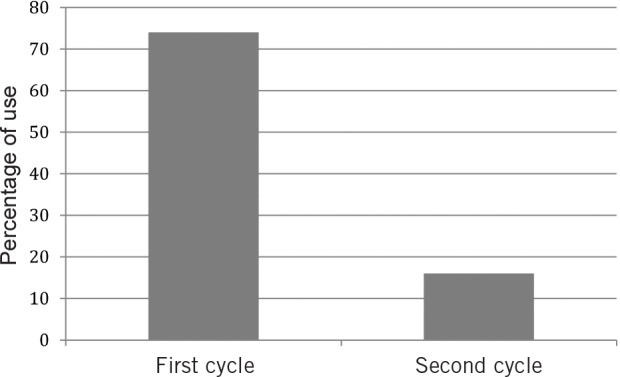
Systemic prophylactic antibiotic use in nasal packing between audit cycles

**Figure 3:**
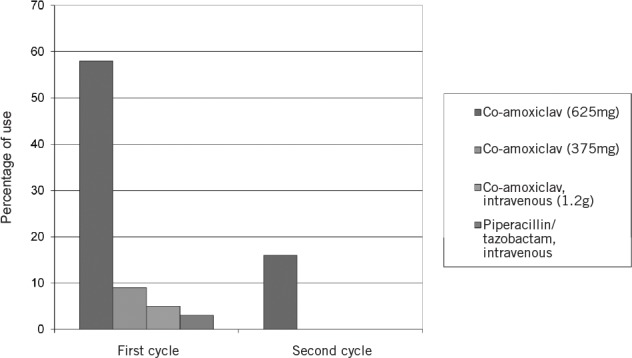
Type and dose of antibiotics used in nasal packing

## Results

A total of 57 patients (35 male, 22 female, mean age: 77 years, range: 11–99 years) were included in the study. The two groups of patients in each audit cycle were well matched in terms of age and sex (*p*>0.05).

Table 1 represents the results of the six-week follow-up questionnaire. Figures 2 and 3 highlight the change in the use of systemic prophylactic antibiotics and the differences in the types of prophylactic antibiotics used prospectively. There was no significant statistical difference in the rates of infective nasal symptoms (crusting, discharge, pain, sinusitis), chest infections, rebleeding or readmission rates to hospital following implementation of these guidelines (*p*>0.05).

## Discussion

In the UK, the practice of prophylactic antibiotic use in nasal packing for spontaneous epistaxis is highly variable. There are currently no published guidelines or randomised controlled trials relating to the use of systemic prophylactic antibiotics in epistaxis patients with nasal packing. **The use of systemic prophylactic antibiotics in nasal packing is an important subject, not only due to the associated resistance development issue but also cost implications.

One study reporting a telephone survey of 71 ENT departments across England found that 22% of departments used no prophylactic systemic antibiotics for anterior nasal packing, 37% used them after 24 hours in situ and 28% did so after 48 hours in situ.^2^ Another study examined the growth of bacteria on packing material following anterior nasal packing in those patients prescribed prophylactic systemic antibiotics and those not receiving any form of antibiotic. No significant difference was found to recommend the routine use of prophylactic systemic antibiotics in anterior nasal packing.^3^

It is hypothesised that nasal packing could cause toxic shock syndrome, a life threatening systemic infection causing multiorgan failure precipitated by infection with either *Staphylococcus aureus* or *Streptococcus pyogenes*, typically occurring with extended tampon use in menstruating women.^4^ In the literature, there are no reported cases of toxic shock syndrome related to anterior nasal packing for acute spontaneous epistaxis. (There are reported cases in packing following nasal surgery.^5^ However, this was not in the remit of this audit.) There was one reported case of endocarditis of a native valve associated with nasal packing; nevertheless, current guidelines on endocarditis prophylaxis do not recommend endocarditis prophylaxis for patients with native heart valves undergoing anterior nasal packing.^6^

The use of systemic antibiotic cover in epistaxis patients with posterior nasal packs is generally accepted practice in the UK.^1^ This is not the case, however, with anterior nasal packs. The aim of this exercise was to establish guidelines for the use of antibiotics in spontaneous epistaxis patients with anterior nasal packs.

After consultation with the literature and local microbiologists, it was agreed, in the absence of any existing infection or other indication for antibiotic use, to use systemic antibiotics only if the anterior packs were in situ for >48 hours and that all patients should receive topical Naseptin^®^ on discharge for a period of 14 days. The use of Naseptin^®^ in all patients following removal of nasal packing is not practised routinely in the UK but it was deemed reasonable following discussion with local microbiologists. In fact, its use may have contributed to decreased levels of recurrent epistaxis and hospital readmission rates following discharge as outlined in the results section.

In the first cycle of the audit, 20 patients (52.6%) requiring anterior nasal packing for ≤48 hours received systemic prophylactic antibiotics (with 74% of all patients receiving prophylactic antibiotics regardless of length of pack insertion). After implementation of the new guidelines, only 15.8% were prescribed systemic prophylactic antibiotics. This represented a 58.2% reduction in routine systemic antibiotic use between cycles. It was evident in the six-week follow-up questionnaire that there were no statistically significant differences between the two cycles in any of the symptom scores or readmission rates for recurrent epistaxis. If anything, there appears to have been a reduction in most symptom scores including rebleeding and readmission rates in the second group although the differences did not reach statistical significance. It is possible that the use of Naseptin^®^ may have actually contributed towards this trend.

### Study limitations

The numbers included in this study, particularly the second cycle of the audit, are admittedly small and this will impact on the power of the study to detect small differences in significant issues such as infective complications. The small numbers are due mainly to limiting inclusion of patients to only those with full datasets. Nevertheless, the study does provide some supporting evidence for this set of guidelines, which is preferable to the existing state of ‘expert opinion’ currently underpinning practice in this area.

## Conclusions

There are currently no validated guidelines for prophylactic antibiotic use in acute epistaxis admissions requiring nasal packing. For this study, a treatment algorithm was introduced to address this. The algorithm has been validated through a complete audit cycle. Its implementation in our department has resulted in a significant reduction in antibiotic use in cases requiring anterior nasal packs without any associated adverse effects, including infection or recurrent epistaxis, necessitating hospital readmission. In the long term, this policy will also help reduce costs and avert issues such as antibiotic resistance. We are currently in the process of running a larger prospective study to further validate the implementation of these guidelines.
